# High-sensitivity cardiac troponin I and frailty: associations with the frailty index and Fried phenotype in older women

**DOI:** 10.1093/gerona/glaf235

**Published:** 2025-10-25

**Authors:** Jedd Pratt, Abadi K Gebre, Carlos J Toro-Huamanchumo, Elsa Dent, Trent Bozanich, Wai E Lim, Elizabeth Byrnes, Julee McDonagh, Caleb Ferguson, Craig Sale, Kun Zhu, Carl Schultz, Richard L Prince, Joshua R Lewis, Marc Sim

**Affiliations:** Department of Sport and Exercise Sciences, Manchester Metropolitan University Institute of Sport, Manchester, United Kingdom; Nutrition and Health Innovation Research Institute, School of Medical and Health Sciences, Edith Cowan University, Perth, Western Australia, Australia; School of Pharmacy, College of Health Sciences, Mekelle University, Mekelle, Tigray, Ethiopia; Nutrition and Health Innovation Research Institute, School of Medical and Health Sciences, Edith Cowan University, Perth, Western Australia, Australia; OBEMET Center for Obesity and Metabolic Health, Lima, Peru; Research Unit for Health Evidence Generation and Synthesis, Universidad San Ignacio de Loyola, Lima, Peru; Institute for Evidence-Based Healthcare, Faculty of Health Sciences & Medicine, Bond University, Queensland, Australia; Nutrition and Health Innovation Research Institute, School of Medical and Health Sciences, Edith Cowan University, Perth, Western Australia, Australia; Nutrition and Health Innovation Research Institute, School of Medical and Health Sciences, Edith Cowan University, Perth, Western Australia, Australia; Department of Renal Medicine, Sir Charles Gairdner Hospital, Perth, Western Australia, Australia; PathWest, QEII Medical Centre, Perth, Western Australia, Australia; School of Nursing, Faculty of Science, Medicine & Health, University of Wollongong, Wollongong, New South Wales, Australia; Centre for Chronic and Complex Care Research, Blacktown Hospital, Western Sydney Local Health District, Blacktown, New South Wales, Australia; School of Nursing, Faculty of Science, Medicine & Health, University of Wollongong, Wollongong, New South Wales, Australia; Centre for Chronic and Complex Care Research, Blacktown Hospital, Western Sydney Local Health District, Blacktown, New South Wales, Australia; Department of Sport and Exercise Sciences, Manchester Metropolitan University Institute of Sport, Manchester, United Kingdom; Department of Endocrinology and Diabetes, Sir Charles Gairdner Hospital, Perth, Western Australia, Australia; Medical School, The University of Western Australia, Perth, Western Australia, Australia; Nutrition and Health Innovation Research Institute, School of Medical and Health Sciences, Edith Cowan University, Perth, Western Australia, Australia; Medical School, The University of Western Australia, Perth, Western Australia, Australia; Nutrition and Health Innovation Research Institute, School of Medical and Health Sciences, Edith Cowan University, Perth, Western Australia, Australia; Medical School, The University of Western Australia, Perth, Western Australia, Australia; Nutrition and Health Innovation Research Institute, School of Medical and Health Sciences, Edith Cowan University, Perth, Western Australia, Australia; Medical School, The University of Western Australia, Perth, Western Australia, Australia

**Keywords:** Frailty, Cardiovascular disease, Biomarkers, Screening, Public health

## Abstract

Despite the nexus between cardiovascular health and frailty, the relevance of high-sensitivity cardiac troponin I (hs-cTnI), a biomarker of myocardial injury, to frailty is poorly understood. We examined whether hs-cTnI concentrations were associated with frailty in a well-characterized cohort of older women. A total of 1151 community-dwelling women from the Perth Longitudinal Study of Aging Women (mean age ± SD = 75.2 ± 2.7 years) were included. Frailty was operationalized using a validated frailty index (FI) of cumulative deficits and a modified Fried phenotype. Plasma hs-cTnI were categorized into quartiles. Cross-sectional associations between hs-cTnI quartiles and frailty were assessed using multivariable-adjusted logistic regression models. A total of 235 (20.4%) women were classified as frail using the FI, while 74 (6.4%) were considered frail by Fried’s phenotype. In a multivariable-adjusted model, compared to women in the lowest hs-cTnI quartile (Q1), those in Q3 and Q4 had 1.38 (95% CI, 1.00-1.90) and 1.79 (1.20-2.67) greater odds for frailty when classified by the FI. When classified according to Fried’s phenotype, women in Q2, Q3, and Q4 had 2.25 (1.10-4.09), 2.64 (1.19-5.21), and 2.44 (1.10-5.33) greater odds for frailty, compared to Q1. Associations remained largely unchanged when further adjusted for daily protein intake or systemic inflammation (lipocalin-2) and restricted to those with subclinical hs-cTnI levels (<15.6ng/L). Higher hs-cTnI levels are associated with greater odds for frailty, classified using an FI or Fried’s phenotype, among older women. hs-cTnI may have applications beyond its typical use in cardiology, offering insight into the implications of underlying cardiovascular dysfunction relating to frailty.

## Introduction

Societal aging will impose an increasingly heavy burden globally, with the proportion of adults aged 65 years or over projected to double, increasing by 1 billion people by 2050.^1^ Among the most burdensome consequences of population aging, on economies, primary care centers, and patients, is the occurrence of geriatric syndromes, such as frailty.^2^^,^^3^ Frailty is often referred to as a multidimensional clinical syndrome characterized by cumulative impairment across several physiological systems (ie, musculoskeletal, neurological, cardiovascular) that reflects the mental (including psychosocial) and physical resilience of an individual.^4^

Although there is no global consensus on a standard frailty classification system, the two most used concepts are the Rockwood and Mitnitski’s cumulative deficit model (ie, frailty index [FI], based upon 30-40 variables spanning several health domains),^5^ and the Fried physical frailty phenotype (ie, based upon 5 pre-defined health variables).^6^ Establishing which concept is most effective for clinical practice has been a topic of significant debate, and although a level of ambiguity remains, both are accepted measures of frailty.^7^ However, the FI is reported to be more effective in identifying individuals at risk of poor health outcomes associated with frailty, given that 20% and 29% of older men and women are typically considered frail by the FI, in comparison to 15% and 11% by a physical frailty phenotype.^8^

Importantly, the presence of frailty, whether determined using an FI or Fried’s phenotype, is associated with increased vulnerability to several adverse health outcomes, including falls and fractures,^9^^,^^10^ physical disability,^10^ hospitalization,^11^ and mortality.^9^^,^^10^ Increasing efforts are being directed to better understand the clinical manifestation of frailty with a view to improve the effectiveness of screening and therapeutic practices. Biomarker research has been proposed as a promising avenue for establishing an accessible means of identifying people with frailty for inclusion in interventions.^4^ Moreover, biomarkers may highlight important physiological mechanisms underpinning the development of frailty, which may allow for the establishment of targeted preventative strategies that focus on maintaining physical and cognitive health.

Cardiovascular health is a central underlying component of frailty, with strong positive associations shown between cardiovascular disease (CVD) and risk of frailty.^12^^,^^13^ Moreover, frailty is prevalent in up to 90% of people living with heart failure with preserved ejection fraction, and consequently, considerable efforts are being directed toward better managing frailty among this patient population.^14^ Therefore, circulating cardiac biomarkers may offer valuable insights into frailty. In particular, cardiac troponin I (cTnI) and cardiac troponin T (cTnT) are well-established markers of myocardial injury, with higher circulating concentrations indicating a greater extent of tissue damage.^15^ Notably, cTnI is primarily expressed by cardiac muscle, and so its circulating concentrations are particularly informative against myocardial injury and are recognized as a clinically relevant indicator of CVD progression.^16^ High-sensitivity cTnI (hs-cTnI) concentrations may be particularly useful for population-based assessments, given that concentrations in the general population are generally much lower than those observed in clinical settings.^15^

Despite the link between CVD and frailty, the relationship between hs-cTnI concentrations and frailty in community-dwelling adults is poorly understood. Existing studies have shown hs-cTnI concentrations to be associated with frailty in older adults with diabetes^17^ and suspected myocardial infarction.^18^ Whether similar observations are recorded in the general population remain unknown. This would be especially relevant to community-dwelling older women who have a particularly high risk of frailty.^8^ Due to the multifactorial etiology of frailty, it is also important to consider whether any potential relationship between hs-cTnI and frailty is independent of other known risk factors, such as systemic inflammation and daily protein intake. For example, chronic systemic inflammation can increase endothelial dysfunction, myocardial injury, and impair physical resilience,^19–22^ while low daily protein intake is associated with reduced muscle mass, strength, and physical function, which are especially pertinent to physical frailty.^23^

Accordingly, the primary aim of this study was to determine whether circulating hs-cTnI concentrations were cross-sectionally associated with frailty, operationalized using a validated FI,^9^ in community-dwelling older women. The secondary aim was to determine whether a similar relationship would be observed using Fried’s phenotype to operationalize frailty.

## Methods

### Participants

Baseline (1998) data from the Perth Longitudinal Study of Ageing Women (PLSAW) were used in the present study. A total of 1500 women from Western Australia who were ≥70 years of age and had an expected survival of 5 years were originally recruited to the Calcium Intake Fracture Outcome Study (CAIFOS), which was a 5-year, double-blind, randomized controlled trial of the effect of calcium supplementation on fracture prevention.^24^ For the present study, 349 women were excluded due to missing data required to compute a FI (*n* = 83), no hs-cTnI measurement (*n* = 250), and missing covariates (socioeconomic position [SEP], *n* = 7; smoking status, *n* = 4; and alcohol intake, *n* = 5). As such, 1151 women were included in this study. Ethical approval was granted by the Human Research Ethics Committee of the University of Western Australia, written informed consent was obtained from each participant, and the study complied with the Declaration of Helsinki. The trial was retrospectively registered at the Australian New Zealand Clinical Trials Registry (#ACTRN12615000750583). Use of data linkage was approved by the Western Australian Department of Health Human Research Ethics Committee (project #2009/24).

### Primary outcome: operationalization of frailty using the FI

Frailty was classified at baseline according to an FI using a standard framework consisting of health deficits, which span several health domains, as described by Searle et al.^25^ These variables include disability in activities of daily living (ADL), instrumental ADL, restricted activity, physical function (eg, impaired walking, impaired grip strength) and general cognition, depression/mood, self-rated health, and co-morbidity. Our FI consisted of 33 variables across these domains, determined through questionnaires such as the short form-36 and Barthel index, in addition to quantitative assessments of body composition, grip strength, timed-up-and-go performance, and blood pressure performed at baseline.^9^ Disease prevalence (eg, coronary heart disease, chronic heart failure, cerebrovascular disease, cancer, diabetes, arthritis, and chronic lung disease) was established via primary discharge diagnoses from 18-year hospital records (1980-1998) obtained through the Western Australian Data Linkage System (Department of Health Western Australia, East Perth, Australia) and the Western Australia Hospital Morbidity Data Collection. International Classification of Diseases-coded diagnosis data were used to reference diseases from public and private inpatient admissions in Western Australia. Each variable was coded with a “1” or a “0,” indicating either the presence or absence of the health variable deficit. To calculate the FI, the total score across variables was calculated and divided by 33, with the final score ranging between 0 and 1. Women were categorized as either non-frail (FI < 0.25) or frail (FI ≥ 0.25) according to previous data.^5^ A full list and corresponding scoring criteria of the variables used to calculate the FI in this cohort have been reported previously.^9^ Notably, our FI identifies women in this cohort who are at increased risk of fall- and fracture-related hospitalization, as well as all-cause mortality.^9^

### High-sensitivity cTnI

Plasma concentrations of hs-cTnI were determined using an Abbott ARCHITECT i2000SR STAT 2-step chemiluminescent microparticle hs-cTnI immunoassay from samples collected at baseline (1998) and stored at −80 °C until analysis in 2013. The assay had a coefficient of variation of 10% at a concentration of 4.7 ng/L and a detection limit of 1.9 ng/L, with some data below this limit being included in the results (*n* = 5; range, 1.4-1.9 ng/L). Plasma hs-cTnI concentrations were categorized into approximate quartiles for data analyses, reducing the impact of extreme values and the skewed nature of the hs-cTnI distribution.

### Assessments

Body mass was measured with a digital scale while participants were dressed lightly and without shoes, and height was measured with a stadiometer. Body mass index (BMI) was calculated as weight divided by height squared (kg/m^2^). Socioeconomic position was determined by matching participants’ residential postcodes with the Australian Bureau of Statistics Index of Relative Socioeconomic Advantage and Disadvantage, which ranked postcodes according to relative socio-economic advantage and disadvantage.^26^ Subsequently, participants were coded into 6 groups, ranging from the top 10% most disadvantaged to the top 10% least disadvantaged. Smoking history was assessed by self-reported questionnaire, with women who had smoked more than 1 cigarette per day for over 3 months at any point in their life classified as either a current smoker or an ex-smoker, depending on current smoking status. Alcohol and protein intake was determined using a validated semi-quantitative food frequency questionnaire (FFQ) established by the Cancer Council of Victoria (DQES V2.0).^27–29^ The FFQ assessed typical frequency of food and alcohol intake over the past 12 months and was completed under the supervision of a research assistant using food models, cups, spoons, and charts to improve accuracy. Plasma lipocalin-2 (LCN2) concentrations were measured using a two-step chemiluminescent microparticle monoclonal immunoassay on an automated platform (Abbott Diagnostics, Longford, Ireland). The inter-assay coefficient of variation over a 6-week period was 9.3% and 4.6% at concentrations of 19 and 190 ng/L, respectively.^30^

### Statistical analyses

Statistical analyses were performed using IBM SPSS Statistics for Windows (V24.0 IBM Corp., Armonk, NY, USA) and R (V3.4.2, R Foundation for Statistical Computing, Vienna, Austria). Participant characteristics were presented according to hs-cTnT quartiles. To allow associations to be non-linear, restricted cubic splines within logistic regression models were used to examine the relationship between hs-cTnI (exposure) and the presence of frailty (outcome, derived from the FI) using the “rms” R package.^31^ Odds ratio (OR) estimates were relative to a reference value being the median hs-cTnI of women with the lowest hs-cTnI concentration quartile (Q1) and were plotted against the respective outcomes with 95% confidence bands provided. Wald tests were used to obtain *p*-values. For visual simplicity only, the *x*-axis was truncated at 2 *SD*s above the mean. For all primary analyses, 2 models of adjustment were adopted including age-adjusted (model 1) and multivariable-adjusted models (model 2) including age, smoking history, SEP, and alcohol intake. Importantly, as the FI includes multiple comorbidities (eg, prevalent CVD, diabetes, arthritis) in addition to physical activity, muscle function (eg, timed-up-and-go, grip strength), and body composition metrics (BMI), these were not added to model 2.

### Further analyses

Given that physical frailty is also commonly assessed in clinical practice, we assessed the relationship between circulating hs-cTnI and a modified Fried phenotype. Typically, the Fried phenotype considers a set of 5 pre-defined domains to identify the absence or presence of frailty,^32^^,^^33^ including (1) unintentional weight loss, (2) muscle weakness, (3) fatigue, (4) slowness, and (5) physical inactivity. If 3 or more of the aforementioned symptoms were present, an individual was classified as frail.^6^ We adopted a modified operational definition of Fried’s phenotype, similar to previous work,^34^ with specific criteria detailed in Table S1.^6^^,^^34^ Subsequently, we assessed the cross-sectional relationship between hs-cTnI concentrations (exposure) and Fried’s phenotype (outcome) using restricted cubic splines within logistic regression models. Considering women in Q4 had higher prevalent CVD, we included prevalent CVD as an additional covariate to model 2 for the primary analysis when considering the Fried phenotype. This was not undertaken for the FI, as clinical CVD contributes to the calculation of the FI.^9^ To examine whether hs‐cTnI concentrations even below the diagnostic threshold for acute coronary syndrome (ACS) were related to frailty, we excluded 50 women with hs-cTnI levels ≥15.6 ng/L.^11^ We re-ran the primary analysis between hs-cTnI and frailty, derived from either an FI or Fried phenotype, using restricted cubic splines within logistic regression models in this sub-group of 1101 women. Although systemic inflammation is a known risk factor for frailty and poor cardiovascular health,^19–22^ previous work suggests that the relationship between hs-cTnT and frailty may be independent of inflammation.^35^ To explore this, we undertook further analyses where LCN2 (also known as neutrophil gelatinase-associated lipocalin; available in 1149 women), a marker of systemic inflammation, was added as an additional covariate to the multivariable-adjusted model when examining the relationship between hs-cTnI and frailty, assessed using the FI as well as Fried’s phenotype. Finally, as a higher protein intake is associated with lower risk for frailty,^23^ we performed additional analyses where total protein intake (g/d; available for all 1151 women) was added to the multivariable-adjusted model.

## Results

**Figure 1.**
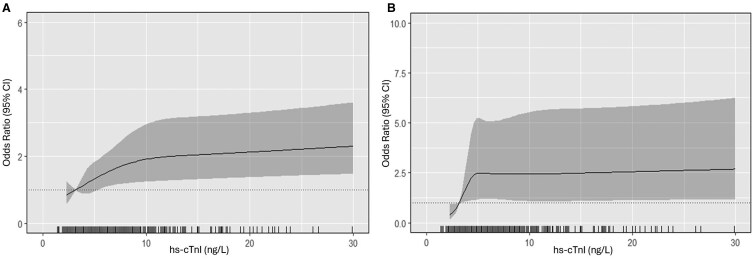
Odds ratios from multivariable-adjusted logistic regression models with restricted cubic spline curves describing the association between high-sensitivity cardiac troponin-I (hs-cTnI) and the presence of frailty, based upon either the Frailty Index (A) or modified Fried phenotype (B). Odds ratios are based on models adjusted for age, smoking history, socioeconomic position, and alcohol intake (model 2). The odds ratio compares hs-cTnI levels (horizontal axis) to the median hs-cTnI level of the lowest quartile (3.2 ng/L). Shading represents 95% confidence regions. The rug plot along the bottom of each graph depicts an observation.

Baseline characteristics of participants according to quartiles of hs-cTnI are presented in Table 1, with the mean ± *SD* age of this cohort being 75.2 ± 2.7 years. Compared to women with the lowest hs-cTnI concentrations (Q1), those with the highest hs-cTnI concentrations (Q4) were slightly older (∼1.3 years), had a higher BMI (∼1.9 kg/m^2^), and prevalence of CVD (21%). The proportion of women identified as frail by the FI was substantially higher in women with the highest hs-cTnI concentrations (Q4; 28.4%) compared to those with the lowest hs-cTnI concentrations (Q1; 16.3%) (Table 2). A diagrammatic representation of the relationship between hs-cTnI and the FI is presented in Figure 1A. Compared to women with the lowest hs-cTnI concentrations (Q1), those with higher hs-cTnI concentrations in Q3 (OR 1.38, 95% CI, 1.00-1.90) and Q4 (OR 1.79, 95% CI, 1.20-2.67) had significantly greater odds for frailty (Table 2).

**Table 1.** Baseline characteristics in all participants by quartiles of high-sensitivity cardiac troponin-I (hs-cTnI).^a^

	All participants	Quartiles of hs-cTnI			
		**Quartile 1** <3.8 ng/L	**Quartile 2** 3.8 to <4.7 ng/L	**Quartile 3** 4.7 to <6.3 ng/L	**Quartile 4** ≥6.3 ng/L
**Number**	1151	307	274	288	282
**Age, years**	75.2 ± 2.7	74.6 ± 2.4	75.0 ± 2.8	75.5 ± 2.7	75.9 ± 2.8
**Body mass index (BMI), kg/m^2^**	27.1 ± 4.7	26.3 ± 4.3	26.4 ± 4.5	27.3 ± 4.5	28.2 ± 5.0
**Smoked ever, yes, *n* (%)**	429 (37.3)	115 (37.5)	98 (35.8)	100 (34.7)	116 (41.1)
**Total protein intake, (g/d)**	76.5 (61.3-94.9)	77.3 (60.9-95.2)	75.0 (60.2-92.7)	77.5 (62.1-95.9)	75.0 (61.5-95.2)
**Alcohol intake, g/d**	1.8 (0.3-10.0)	2.2 (0.3-11.6)	2.2 (0.3-10.7)	1.3 (0.2-8.9)	1.0 (0.2-8.8)
***Socioeconomic position, n (%)***					
**Top 10% most highly disadvantaged**	53 (4.6)	10 (3.3)	17 (6.2)	16 (5.6)	10 (3.6)
**Highly disadvantaged**	137 (11.9)	31 (10.1)	31 (11.3)	39 (13.5)	36 (12.8)
**Moderate-highly disadvantaged**	188 (16.3)	55 (17.9)	40 (14.6)	41 (14.2)	52 (18.4)
**Low-moderately disadvantaged**	181 (15.7)	47 (15.3)	41 (15.0)	54 (18.8)	39 (13.8)
**Low disadvantaged**	243 (21.1)	56 (18.2)	70 (25.6)	57 (19.8)	60 (21.3)
**Top 10% least disadvantaged**	349 (30.3)	108 (35.2)	75 (27.4)	81 (28.1)	85 (30.1)
**Prevalent cardiovascular disease, yes, *n* (%)**	269 (23.4)	53 (17.3)	51 (18.6)	57 (19.8)	108 (38.3)
**Prevalent diabetes, yes, *n* (%)**	67 (5.8)	16 (5.2)	7 (2.6)	15 (5.2)	29 (10.3)
**hs-cTnI, ng/L**	4.6 (3.7-6.2)	3.2 (2.8-3.5)	4.2 (4.0-4.4)	5.3 (4.9-5.7)	8.5 (6.9-12.3)
**LCN2, mg/L** ^b^	76.5 (62.0-95.1)	71.8 (58.5-90.0)	73.8 (60.9-91.2)	79.2 (64.5-99.9)	82.1 (67.2-102.1)
**Timed-up-and-go, s**	9.5 (8.2-11.1)	9.1 (8.1-10.6)	9.3 (8.1-10.5)	9.5 (8.3-11.2)	10.3 (8.8-11.9)
**Handgrip strength, kg**	20.4 (4.8)	20.7 (4.7)	20.5 (5.1)	20.3 (4.9)	20.0 (4.7)

Abbreviation: LCN2, lipocalin-2.

a Data presented as mean ± SD, median (interquartile range) or *n* (%) where appropriate.

b *n* = 1149.

**Table 2.** Odds ratio (95% CI) for the presence of frailty, based upon either the Frailty Index (FI) or modified Fried phenotype, by quartiles of high-sensitivity cardiac troponin-I (hs-cTnI).

	Quartiles of hs-cTnI^a^			
	**Quartile 1** <3.8 ng/L	**Quartile 2** 3.8 to <4.7 ng/L	**Quartile 3** 4.7 to <6.3 ng/L	**Quartile 4** ≥6.3 ng/L
***FI, n (%)***	50 (16.3)	44 (16.1)	61 (21.2)	80 (28.4)
***Model 1***	Ref.	1.22 (0.92-1.63)	**1.46 (1.06-2.00)**	**1.94 (1.33-2.91)**
***Model 2***	Ref.	1.18 (0.88-1.58)	**1.38 (1.00-1.90)**	**1.79 (1.20-2.67)**
***Fried phenotype, n (%)***	12 (3.9)	16 (5.8)	20 (6.9)	26 (9.2)
***Model 1***	Ref.	**2.22 (1.16-4.27)**	**2.68 (1.29-5.58)**	**2.71 (1.25-5.88)**
***Model 2***	Ref.	**2.25 (1.15-4.38)**	**2.64 (1.25-5.59)**	**2.44 (1.14-5.52)**

a Estimated odds and 95% CI from logistic regression analysis comparing the median hs-cTnI level from each quartile (Q) compared to Q1. Median Q1, Q2, Q3, and Q4 for hs-cTnI was 3.2, 4.2, 5.3, and 8.5 ng/L, respectively. Model 1: adjusted for age. Model 2: model 1 + smoking history, socioeconomic position, and alcohol intake. Bolded indicates *p* < .05 compared to Q1.

### Further analyses

Physical frailty was identified in 74 (6.4%) women, which was substantially lower than when frailty was identified using the FI (*n* = 235, 20.4%). A diagrammatic representation of the relationship between hs-cTnI and physical frailty is presented in Figure 1B. Compared to women with the lowest hs-cTnI concentrations (Q1), those with higher hs-cTnI concentrations in Q2 (OR: 2.25, 95% CI, 1.15-4.38), Q3 (OR: 2.64, 95% CI, 1.25-5.59), and Q4 (OR: 2.44, 95% CI, 1.14-5.52) had significantly greater odds for physical frailty in the multivariable-adjusted analysis (Table 2). When prevalent CVD was included as an additional covariate to model 2, compared to Q1, the relationship between hs-cTnI and the Fried phenotype remained comparable (Q2: OR: 2.12, 95% CI, 1.10-4.11; Q3: OR: 2.47, 95% CI, 1.17-5.18; and Q4: OR: 2.35, 95% CI, 1.07-5.18).

After excluding 50 individuals with clinically elevated hs-cTnI concentrations, the proportion of women identified as frail by the FI was 19.7%, with the greatest proportion being recorded in women with the highest hs-cTnI concentration (Q4; 25.6%). The proportion of women identified as frail by the Fried phenotype was 6.4%, with the greatest proportion again being shown in women with the highest hs-cTnI concentration (Q4; 10.3%). A diagrammatic representation of the relationship between hs-cTnI levels (below the clinical threshold) and frailty, assessed using either the FI or Fried phenotype, is presented in Figure S1A and B, respectively. When considering the FI, compared to women with the lowest hs-cTnI concentrations (Q1), those with higher hs-cTnI concentrations in Q3 (OR: 1.49, 95% CI, 1.05-2.12) and Q4 (OR: 1.93, 95% CI, 1.25-2.98) had greater odds for frailty in the age-adjusted analysis (Table S2). In the multivariable-adjusted model, greater odds for frailty were only observed when comparing women with the highest (Q4; OR: 1.79, 95% CI, 1.16-2.77) compared to the lowest hs-cTnI concentration (Q1). When considering Fried’s phenotype, women in Q2 (OR: 2.18, 95% CI, 1.03-4.60), Q3 (OR: 2.95, 95% CI, 1.32-6.60), and Q4 (OR: 3.36, 95% CI: 1.46-7.72) of hs-cTnI levels had greater odds for physical frailty in the age-adjusted analysis, compared to individuals with the lowest levels (Q1) (Table S2). In the multivariable-adjusted model, greater odds for physical frailty were shown in women in Q3 (OR: 2.73, 95% CI, 1.22-6.12) and Q4 (OR: 3.09, 95% CI, 1.34-7.13), compared to women with the lowest hs-cTnI levels (Q1).

When LCN2 was added to the multivariable-adjusted analysis, only individuals with the highest hs-cTnI levels (Q4: OR: 1.67, 95% CI, 1.12-2.50) recorded higher odds for frailty, classified by FI, when compared to those with the lowest hs-cTnI levels (Q1) (Table S3). When considering Fried’s phenotype, individuals in Q2 (OR: 2.06, 95% CI, 1.04-3.96) and Q3 (OR: 2.34, 95% CI, 1.12-4.89) of hs-cTnI levels recorded greater odds for frailty, when compared to the lowest hs-cTnI levels (Q1). This relationship was no longer statistically significant in those with the highest hs-cTnI levels (Q4; OR: 2.16, 95% CI, 0.98-4.75) (Table S3). When protein intake was added to the multivariable-adjusted model, women in Q3 (OR: 1.38, 95% CI, 1.00-1.90) and Q4 (OR: 1.80, 95% CI, 1.21-2.68) of hs-cTnI levels showed greater odds for frailty, classified by FI, compared to women with the lowest hs-cTnI levels (Q1) (Table S4). When classified by Fried’s phenotype, women in Q2 (OR: 2.11, 95% CI, 1.09-4.07), Q3 (OR: 2.48, 95% CI, 1.18-5.19), and Q4 (OR: 2.44, 95% CI, 1.12-5.34) of hs-cTnI levels showed greater odds for frailty compared to women with the lowest hs-cTnI levels (Q1) (Table S4).

## Discussion

This study demonstrates that higher hs-cTnI concentrations are associated with increased odds for frailty among community-dwelling older women. Specifically, women with higher circulating hs-cTnI concentrations (Q3 and Q4) were 38%-79% more likely to be frail, classified by FI, than women within the lowest hs-cTnI quartile. Notably, the associations between hs-cTnI and frailty were present regardless of whether frailty was operationalized according to an FI or Fried’s phenotype. Indeed, when classified by Fried’s phenotype, women with higher circulating hs-cTnI concentrations (Q2-Q4) were 125%-164% more likely to be frail, than women within the lowest hs-cTnI quartile. Notably, these associations were evident at hs-cTnI concentrations far below the diagnostic threshold for ACS. In fact, there were clear plateaus in the associations between hs-cTnI and frailty, at 10 ng/L for the FI, and 5 ng/L for Fried’s phenotype, further supporting the relevance of sub-clinical hs-cTnI as a biomarker of frailty in older community-dwelling women.

Until now, the relationship between cardiac troponins and frailty has been largely based on hs‑cTnT.^17^^,^^36–38^ Our findings strengthen existing literature supporting the nexus between cardiac health and frailty, while also highlighting the potential role of hs‑cTnI in frailty research. The few available studies relating to hs-cTnI have focused on cohorts with comorbidities (ie, diabetics or suspected myocardial infarction patients),^17^^,^^18^ and as such, their findings may not be transferable to the general population. This is especially relevant as such populations are more likely to present with frailty, especially when assessed using an FI. Our study is the first to demonstrate strong positive associations between circulating hs-cTnI and frailty, irrespective of whether frailty was assessed using a multidimensional FI or Fried’s phenotype. We also show that the associations between hs-cTnI and frailty are robust to adjustment for daily protein intake, a known risk factor for frailty.^35^

Although the physiological mechanisms underpinning the observed associations are not fully understood, a few plausible hypotheses exist. For example, chronic inflammation is a strong risk factor for endothelial dysfunction and myocardial injury,^19^^,^^20^ as well as reduced skeletal muscle mass and physical resilience.^21^^,^^22^ We found that the relationship between hs-cTnI and frailty derived from the FI remained comparable when a marker of inflammation, LCN2, was added to our analysis. When considering physical frailty, statistical significance was only attenuated in women with the highest hs-cTnI levels (Q4), but not in Q3, after adjustment of LCN2, indicating a potential influence of inflammation. Such findings tend to support previous work in older men reporting comparable relationships between hs-cTnT, inflammatory biomarkers (ie, C-reactive protein [CRP] and interleukin-6 [IL-6]), and physical frailty.^34^ Future studies should build upon these data by incorporating a broader panel of inflammatory biomarkers, such as LCN2, IL-6, CRP, and TNF-α, to better delineate the potential mediating effect of inflammation on the association between hs-cTnI and frailty. In addition to inflammation, elevated hs-cTnI may also reflect microvascular dysfunction and myocardial remodeling.^38^ Such changes may contribute toward low-grade cardiac damage, progressively reducing cardiovascular reserve and the individual’s overall physiological resilience over time. Clearly, such hypotheses warrant further investigation.

Associations between circulating hs-cTnI and different frailty classifications (ie, the FI and Fried’s phenotype) are particularly noteworthy, as they demonstrate the implications of underlying cardiac, vascular, and metabolic abnormalities, as reflected by elevated hs-cTnI levels, on frailty in older women. This supports the relevance of circulating hs-cTnI to multiple dimensions of frailty, encompassing both the systemic physiological decline captured by the FI and the physical and functional decline captured by Fried’s phenotype. Given the lack of harmonization among existing frailty definitions, identifying biomarkers that reflect shared physiological pathways may advance our understanding of the biological underpinnings of frailty, particularly in the context of CVD.^4^ Notably, irrespective of how frailty might be characterized (ie, FI or physical frailty phenotype), the Australian Cardiovascular Alliance National Taskforce on the Management of Frailty in Heart Failure also recently raised the importance of identifying clinically relevant biomarkers that may be especially useful in understanding potential biological mechanisms.^14^ Such biomarkers may also be useful when assessing treatment efficacy and/or developing prevention strategies for frailty.^39^ Indeed, our findings provide evidence that hs-cTnI may have value beyond its typical use in diagnosing ACS or aiding CVD risk stratification,^40^ offering insights into the role that cardiac stress and systemic dysfunction, may have in the development of frailty.

We observed plateaus in the strength of the association between frailty and pre-clinical hs-cTnI levels at approximately 10 ng/L for the FI and 5 ng/L for Fried’s phenotype, providing potential thresholds for future studies considering incident frailty. This is an important consideration due to the cross-sectional design of our study, which limits any inference regarding temporal associations between hs-cTnI and frailty. Longitudinal studies should also examine if changes in hs-cTnI over time are related to frailty progression. Notably, evidence from existing longitudinal studies supports a temporal relationship between cardiac troponins and frailty, with higher baseline hs-cTnT concentrations shown to be associated with incident frailty among community-dwelling older men and women.^36^^,^^41^ Interestingly, increases in circulating hs-cTnT over a 6-year period have also been linked with incident frailty assessed 15 years later.^36^ There is also evidence that higher baseline concentrations of N-terminal pro B-type natriuretic peptide, a biomarker of cardiac strain, is associated with incident frailty among community-dwelling populations,^36^ although conflicting findings have been shown.^41^ Whether a similar pattern is shown for hs-cTnI and incident frailty remains to be determined. From a clinical perspective, the application of our findings may lie less in using hs-cTnI as a screening tool and more in recognizing its role as a marker of underlying cardiac dysfunction contributing to frailty. An important next step may be to determine whether interventions that improve frailty status, such as exercise or nutrition, also lead to reductions in hs-cTnI levels. Given that interventions targeting frailty can reduce systemic inflammatory markers, albeit not consistently,^42^ it is plausible that improving frailty status may lower CVD risk and, therefore, reduce circulating hs-cTnI. If so, hs-cTnI may offer value as a marker of intervention efficacy, particularly in the context of integrated care models targeting both frailty and cardiovascular health in older populations.

The main strength of this study is the large, well-characterized cohort of community-dwelling older women, a population with a particularly high risk for frailty.^8^ This is demonstrated in our findings, where ∼1 in 5 women were classified as frail based upon the FI, despite recruitment criteria requiring participants to be generally healthy, free from major disease, and with an estimated survival beyond 5 years.^24^ As such, even within a relatively healthy cohort of older women, those with slightly elevated hs-cTnI levels (>4.7 ng/L) presented with significantly greater odds for frailty, demonstrating the strong interplay between cardiac health and frailty in this population. Furthermore, frailty was assessed using both the FI and Fried’s phenotype, 2 of the most common operational concepts of frailty. There are several limitations to acknowledge, the most important of which is the cross-sectional design of this observational study, which precludes the establishment of causality and could be affected by residual confounding. As a longitudinal measure of the FI is not available in this cohort, there remains a need to assess the associations prospectively. Although the present study focused solely upon older White women, known sex- and ethnic-specific differences in frailty and hs-cTnI warrant further investigation. For example, in the general population, women generally have lower hs-cTnI levels than men, yet its prognostic value for cardiovascular events is stronger among women.^43^^,^^44^ Ethnic differences have also been reported with White men and women having higher hs-cTnI concentrations than South Asian populations.^45^ Regarding frailty, beyond its well-established higher incidence among women compared to men,^8^ ethnic differences have also been shown, with Black and South Asian populations exhibiting higher incidence and progression of frailty in comparison to White populations.^46^ Collectively, these data highlight the need for future studies integrating more diverse cohorts to determine the extent by which our findings can be generalized across sex and ethnic groups. Finally, it is possible that the higher prevalence of CVD in the highest hs-cTnI quartile may confound the observed associations between frailty and hs-cTnI. To examine this further, supplementary analyses were undertaken, demonstrating that the associations with both measures of frailty persisted even when women with clinically elevated hs-cTnI concentrations (≥15.6 ng/L) were excluded. Additionally, when adjusted for prevalent CVD, the relationship between hs-cTnI and Fried’s phenotype remained comparable. Combined, these data suggest that the link between hs-cTnI and frailty may, in part, be driven by chronic lower-grade myocardial stress or injury. This is consistent with existing evidence suggesting that frailty and cardiovascular multimorbidity are independently associated with elevated hs-cTnI.^18^

In conclusion, higher hs-cTnI concentrations are associated with greater odds for frailty, assessed using either an FI or Fried’s phenotype, in community-dwelling older women. Our findings show that hs-cTnI may have applications beyond its conventional use in cardiology. Going forward, examining whether hs-cTnI levels respond to clinical intervention, such as lifestyle modification and exercise, may improve our understanding of the relationship between cardiac troponins, frailty, and therapeutic strategies, and ultimately help to optimize programs to support cardiovascular health and physical resilience among older populations.

## Supplementary Material

glaf235_Supplementary_Data

## References

[1] Sander M , Oxlund B, Jespersen A, et al The challenges of human population ageing. Age Ageing. 2015;44:185-187. 10.1093/ageing/afu189 25452294 PMC4339729

[2] Prince MJ , Wu F, Guo Y, et al The burden of disease in older people and implications for health policy and practice. Lancet. 2015;385:549-562. 10.1016/s0140-6736(14)61347-7 25468153

[3] Hoogendijk EO , Afilalo J, Ensrud KE, Kowal P, Onder G, Fried LP. Frailty: implications for clinical practice and public health. Lancet. 2019;394:1365-1375. 10.1016/s0140-6736(19)31786-6 31609228

[4] Dent E , Hanlon P, Sim M, et al Recent developments in frailty identification, management, risk factors and prevention: a narrative review of leading journals in geriatrics and gerontology. Ageing Res Rev. 2023;91:102082. 10.1016/j.arr.2023.102082 37797723

[5] Rockwood K , Mitnitski A. Frailty in relation to the accumulation of deficits. J Gerontol A Biol Sci Med Sci. 2007;62:722-727. 10.1093/gerona/62.7.722 17634318

[6] Fried LP , Tangen CM, Walston J, et al Frailty in older adults: evidence for a phenotype. J Gerontol A Biol Sci Med Sci. 2001;56:M146-M157. 10.1093/gerona/56.3.m146 11253156

[7] Dent E , Kowal P, Hoogendijk EO. Frailty measurement in research and clinical practice: a review. Eur J Intern Med. 2016;31:3-10. 10.1016/j.ejim.2016.03.007 27039014

[8] O'Caoimh R , Sezgin D, O'Donovan MR, et al Prevalence of frailty in 62 countries across the world: a systematic review and meta-analysis of population-level studies. Age Ageing. 2021;50:96-104. 10.1093/ageing/afaa219 33068107

[9] Dent E , Dalla Via J, Bozanich T, et al Frailty increases the long-term risk for fall and fracture-related hospitalizations and all-cause mortality in community-dwelling older women. J Bone Miner Res. 2024;39:222-230. 10.1093/jbmr/zjad019 38477757 PMC11240159

[10] Ensrud KE , Ewing SK, Cawthon PM, et al A comparison of frailty indexes for the prediction of falls, disability, fractures, and mortality in older men. J Am Geriatr Soc. 2009;57:492-498. 10.1111/j.1532-5415.2009.02137.x 19245414 PMC2861353

[11] Chang SF , Lin HC, Cheng CL. The relationship of frailty and hospitalization among older people: evidence from a meta-analysis. J Nurs Scholarsh. 2018;50:383-391. 10.1111/jnu.12397 29874399

[12] James K , Jamil Y, Kumar M, et al Frailty and cardiovascular health. J Am Heart Assoc. 2024;13:e031736. 10.1161/JAHA.123.031736 39056350 PMC11964060

[13] Frisoli A , Ingham SJM, Paes ÂT, et al Frailty predictors and outcomes among older patients with cardiovascular disease: data from Fragicor. Arch Gerontol Geriatr. 2015;61:1-7. 10.1016/j.archger.2015.03.001 25921097

[14] McDonagh J , Ferguson C, Hilmer SN, et al An expert opinion on the management of frailty in heart failure from the Australian Cardiovascular Alliance National Taskforce. Heart Lung Circ. 2025;34:693-703. 10.1016/j.hlc.2025.01.012 40107957

[15] Welsh P , Preiss D, Hayward C, et al Cardiac troponin T and troponin I in the general population. Circulation. 2019;139:2754-2764. 10.1161/CIRCULATIONAHA.118.038529 31014085 PMC6571179

[16] Samman Tahhan A , Sandesara P, Hayek SS, et al High‐sensitivity troponin I levels and coronary artery disease severity, progression, and long‐term outcomes. J Am Heart Assoc. 2018;7:e007914. 10.1161/JAHA.117.007914 29467150 PMC5866331

[17] Tang O , Daya N, Matsushita K, et al Performance of high-sensitivity cardiac troponin assays to reflect comorbidity burden and improve mortality risk stratification in older adults with diabetes. Diabetes Care. 2020;43:1200-1208. 10.2337/dc19-2043 32161049 PMC7245347

[18] Ticinesi A , Nouvenne A, Cerundolo N, et al Accounting for frailty and multimorbidity when interpreting high-sensitivity troponin I tests in oldest old. J Am Geriatr Soc. 2022;70:549-559. 10.1111/jgs.17566 34792185 PMC9299120

[19] Theofilis P , Sagris M, Oikonomou E, et al Inflammatory mechanisms contributing to endothelial dysfunction. Biomedicines. 2021;9:781. 10.3390/biomedicines9070781 34356845 PMC8301477

[20] Claesson-Welsh L , Dejana E, McDonald DM. Permeability of the endothelial barrier: identifying and reconciling controversies. Trends Mol Med. 2021;27:314-331. 10.1016/j.molmed.2020.11.006 33309601 PMC8005435

[21] Tuttle CSL , Thang LAN, Maier AB. Markers of inflammation and their association with muscle strength and mass: a systematic review and meta-analysis. Ageing Res Rev. 2020;64:101185. 10.1016/j.arr.2020.101185 32992047

[22] Pellegrino R , Paganelli R, Di Iorio A, et al Lack of immune resilience negatively affects physical resilience: results from the InCHIANTI follow-up study. J Gerontol A Biol Sci Med Sci. 2024;79:glae076. 10.1093/gerona/glae076 38457361 PMC11003532

[23] Struijk EA , Fung TT, Rodríguez-Artalejo F, et al Protein intake and risk of frailty among older women in the Nurses’ Health Study. J Cachexia Sarcopenia Muscle. 2022;13:1752-1761. 10.1002/jcsm.12972 35318829 PMC9178161

[24] Prince RL , Devine A, Dhaliwal SS, Dick IM. Effects of calcium supplementation on clinical fracture and bone structure: results of a 5-year, double-blind, placebo-controlled trial in elderly women. Arch Intern Med. 2006;166:869-875. 10.1001/archinte.166.8.869 16636212

[25] Searle SD , Mitnitski A, Gahbauer EA, Gill TM, Rockwood K. A standard procedure for creating a frailty index. BMC Geriatr. 2008;8:24. 10.1186/1471-2318-8-24 18826625 PMC2573877

[26] Australian Bureau of Statistics. *Census of Population and Housing: socio-Economic Indexes for Areas*. Catalogue no. 2039.0. Australian Bureau of Statistics; 1991.

[27] Hodge A , Patterson AJ, Brown WJ, Ireland P, Giles G. The Anti Cancer Council of Victoria FFQ: relative validity of nutrient intakes compared with weighed food records in young to middle-aged women in a study of iron supplementation. Aust N Z J Public Health. 2000;24:576-583. 10.1111/j.1467-842x.2000.tb00520.x 11215004

[28] Ireland P , Jolley D, Giles G, et al Development of the Melbourne FFQ: a food frequency questionnaire for use in an Australian prospective study involving an ethnically diverse cohort. Asia Pac J Clin Nutr. 1994;3:19-31. 24351203

[29] Woods RK , Stoney RM, Ireland PD, et al A valid food frequency questionnaire for measuring dietary fish intake. Asia Pac J Clin Nutr. 2002;11:56-61. 10.1046/j.1440-6047.2002.00273.x 11892722

[30] Bauer C , Sim M, Prince RL, et al Circulating lipocalin-2 and features of metabolic syndrome in community-dwelling older women: a cross-sectional study. Bone. 2023;176:116861. 10.1016/j.bone.2023.116861 37524293

[31] Harrell F. rms: Regression Modeling Strategies. R package version 5.1-3.1. Accessed February 2025. https://hbiostat.org/r/rms/

[32] Dent E , Morley J, Cruz-Jentoft A, et al Physical frailty: ICFSR international clinical practice guidelines for identification and management. J Nutr Health Aging. 2019;23:771-787. 10.1007/s12603-019-1273-z 31641726 PMC6800406

[33] Cesari M , Gambassi G, Abellan van Kan G, Vellas B. The frailty phenotype and the frailty index: different instruments for different purposes. Age Ageing. 2014;43:10-12. 10.1093/ageing/aft160 24132852

[34] Thompson MQ , Theou O, Karnon J, Adams RJ, Visvanathan R. Frailty prevalence in Australia: findings from four pooled Australian cohort studies. Australas J Ageing. 2018;37:155-158. 10.1111/ajag.12483 29314622

[35] McKechnie DGJ , Patel M, Papacosta AO, et al Associations between inflammation, coagulation, cardiac strain and injury, and subclinical vascular disease with frailty in older men: a cross-sectional study. BMC Geriatrics. 2022;22:405. 10.1186/s12877-022-03106-3 35527242 PMC9082861

[36] Jia Y , Li D, Yu J, et al Subclinical cardiovascular disease and frailty risk: the atherosclerosis risk in communities study. BMC Geriatrics. 2022;22:321. 10.1186/s12877-022-02974-z 35413794 PMC9006603

[37] Strandberg LS , Roos A, Holzmann MJ. Stable high-sensitivity cardiac troponin T levels and the association with frailty and prognosis in patients with chest pain. Am J Med Open. 2021;1-6:100001. 10.1016/j.ajmo.2021.100001 39036625 PMC11256254

[38] Quesada O , Elboudwarej O, Nelson MD, et al Ultra-high sensitivity cardiac troponin-I concentration and left ventricular structure and function in women with ischemia and no obstructive coronary artery disease. Am Heart J Plus. 2022;13:100115. 10.1016/j.ahjo.2022.100115 35784010 PMC9246284

[39] Rodriguez-Mañas L , Araujo de Carvalho I, Bhasin S, et al ICFSR task force perspective on biomarkers for sarcopenia and frailty. J Frailty Aging. 2020;9:4-8. 10.14283/jfa.2019.32 32150207 PMC12275745

[40] Marston NA , Bonaca MP, Jarolim P, et al Clinical application of high-sensitivity troponin testing in the atherosclerotic cardiovascular disease framework of the current cholesterol guidelines. JAMA ­Cardiol. 2020;5:1255-1262. 10.1001/jamacardio.2020.2981 PMC7407328 32756916

[41] McKechnie DGJ , Papacosta AO, Lennon LT, Ramsay SE, Whincup PH, Wannamethee SG. Associations between inflammation, cardiovascular biomarkers and incident frailty: the British Regional Heart Study. Age Ageing. 2021;50:1979-1987. 10.1093/ageing/afab143 34254997 PMC8675445

[42] Byrne T , Cooke J, Bambrick P, McNeela E, Harrison M. Circulating inflammatory biomarker responses in intervention trials in frail and sarcopenic older adults: a systematic review and meta-analysis. Exp Gerontol. 2023;177:112199. 10.1016/j.exger.2023.112199 37156445

[43] Kimenai DM , Shah ASV, McAllister DA, et al Sex differences in cardiac troponin I and T and the prediction of cardiovascular events in the general population. Clin Chem. 2021;67:1351-1360. 10.1093/clinchem/hvab109 34240125 PMC8486023

[44] de Bakker M , Anand A, Shipley M, et al Sex differences in cardiac troponin trajectories over the life course. Circulation. 2023;147:1798-1808. 10.1161/circulationaha.123.064386 37114498 PMC10249606

[45] Kalaria TR , Harris N, Sensi H, et al High-sensitivity cardiac troponin I: is ethnicity relevant? J Clin Pathol. 2021;74:709-711. 10.1136/jclinpath-2020-206951 33782194

[46] Khan M , Nicholl BI, Hanlon P. Ethnicity and frailty: a systematic review of association with prevalence, incidence, trajectories and risks. Ageing Res Rev. 2025;109:102759. 10.1016/j.arr.2025.102759 40306389

