# Supplementary material for: High-sensitivity cardiac troponin I and frailty: associations with the frailty index and Fried phenotype in older women
Source: J Gerontol A Biol Sci Med Sci. 2025 Oct 25;81(2):glaf235. doi: 10.1093/gerona/glaf235 (PMC12815600; doi:10.1093/gerona/glaf235)
Supplement: glaf235_Supplementary_Data [file glaf235_supplementary_data.docx]

**Supplementary Material for ‘High-Sensitivity Cardiac Troponin I and Frailty: Associations with the Frailty Index and Fried Phenotype in Older Women’**

**Contents**

**Table S1.** A comparison of the criteria between the original and modified Fried frailty phenotype adopted.

**Table S2.** Odds ratio (95%CI) for the presence of frailty, derived from either the Frailty Index (FI) or modified Fried phenotype, by quartiles of high-sensitivity cardiac troponin-I (hs-cTnI) in 1,101 individuals with hs-cTnI <15.6 ng/L.

**Table S3.** Odds ratio (95%CI) for the presence of frailty, derived from either the Frailty Index (FI) or modified Fried phenotype, by quartiles of high-sensitivity cardiac troponin-I (hs-cTnI) in the multivariable-adjusted model with the addition of lipocalin-2 (LCN2).

**Table S4.** Odds ratio (95%CI) for the presence of frailty, derived from either the Frailty Index (FI) or modified Fried phenotype, by quartiles of high-sensitivity cardiac troponin-I (hs-cTnI) in the multivariable-adjusted model with the addition of total protein intake (g/d).

**Figure S1.** Odds ratios from multivariable-adjusted logistic regression models with restricted cubic spline curves describing the association between high-sensitivity cardiac troponin-I (hs-cTnI) and the presence of frailty, based upon either the Frailty Index (A) or modified Fried phenotype (B), in 1101 individuals with hs-cTnI <15.6 ng/L. Odds ratios are based on models adjusted for age, smoking history, socioeconomic position and alcohol intake (Model 2). The odds ratio compares hs-cTnI levels (horizontal axis) to the median hs-cTnI level of the lowest quartile (3.1 ng/L). Shading represents 95% confidence regions. The rug plot along the bottom of each graph depicts an observation

| **Table S1**. A comparison of the criteria between the original and modified Fried frailty phenotype adopted. | | |
| --- | --- | --- |
| **Frailty domain** | **Original Fried physical frailty phenotype** | **Modified Fried physical frailty phenotype** |
| Weight loss | Unintentional weight loss of ≥10 pounds in prior year or at follow-up, of ≥5% of body weight in prior year (by direct measurement of weight) | Body mass index <21 kg/m^2^ during participants clinical visit |
| Weakness | Grip strength (kg) measured using dynamometer in the lowest 20% at baseline adjusted for sex and BMI. Cut points stratified by sex and BMI | Grip strength (kg) using dynamometer in the lowest 20% at baseline, adjusting for body mass index (<0.5978 kg/BMI in kg/m^2^) |
| Exhaustion | Two questions used from the CES–D Scale. How often in the last week did you feel: (i) I felt that everything I did was an effort; and (ii) I could not get going Scoring: Rarely or none (0), some or a little (1), moderate (2), most (3). Deficit present when answering ‘2’ or ‘3’ to either question | Responding to the SF36 item “How much of the time during the past 4 weeks did you feel worn out?” with “all of the time”, “most of the time” or “good bit of the time” |
| Slowness | The slowest 20% of the population based on time to walk 15 feet, adjusting for sex and height | The slowest 20% of the population based on time to rise from chair, walk 3 metres, turn, return to sit on chair, adjusting for height. (>10.0727 seconds/height in m) |
| Low physical activity | Minnesota Leisure Time Activity Questionnaire. Kilocalories per week stratified by sex | Performed 0 Kcal of physical activity per day |
| CES-D, Centre for Epidemiologic Studies Depression; SF36, 36-item Short Form Survey. Table is adapted from Thompson et al. 2018 [29] | | |

| **Table S2.** Odds ratio (95%CI) for the presence of frailty, derived from either the Frailty Index (FI) or modified Fried phenotype, by quartiles of high-sensitivity cardiac troponin-I (hs-cTnI) in 1,101 individuals with hs-cTnI <15.6 ng/L. | | | | |
| --- | --- | --- | --- | --- |
|  | **Quartiles of hs-cTnI^1^** | | | |
|  | **Quartile 1**  <3.6 ng/L | **Quartile 2**  3.6 to <4.5 ng/L | **Quartile 3**  4.5 to <5.9 ng/L | **Quartile 4**  ≥5.9 ng/L |
| *FI, n (%)* | 36 (14.7) | 45 (16.4) | 64 (21.3) | 72 (25.6) |
| *Model 1* | Ref. | 1.21 (0.88-1.68) | **1.49 (1.05-2.12)** | **1.93 (1.25-2.98)** |
| *Model 2* | Ref. | 1.16 (0.83-1.61) | 1.40 (0.98-2.00) | **1.79 (1.16-2.77)** |
| *Fried phenotype, n (%)* | 7 (2.9) | 12 (4.4) | 22 (7.3) | 29 (10.3) |
| *Model 1* | Ref. | **2.18 (1.03-4.60)** | **2.95 (1.32-6.60)** | **3.36 (1.46-7.72)** |
| *Model 2* | Ref. | 2.03 (0.96-4.30) | **2.73 (1.22-6.12)** | **3.09 (1.34-7.13)** |
| ^1^Estimated odds and 95%CI from logistic regression analysis comparing the median hs-cTnI level from each quartile (Q) compared to Q1. Median Q1, Q2, Q3 and Q4 for hs-cTnI was 3.1, 4.1, 5.1 and 7.3 ng/L, respectively. Model 1: adjusted for age. Model 2: Model 1 + smoking history, socioeconomic position and alcohol intake. Bolded indicates p<0.05 compared to Q1. | | | | |

| **Table S3.** Odds ratio (95%CI) for the presence of frailty, derived from either the Frailty Index (FI) or modified Fried phenotype, by quartiles of high-sensitivity cardiac troponin-I (hs-cTnI) in the multivariable-adjusted model with the addition of lipocalin-2 (LCN2). | | | | |
| --- | --- | --- | --- | --- |
|  | **Quartiles of hs-cTnI^1^** | | | |
|  | **Quartile 1**  <3.7 ng/L | **Quartile 2**  3.7 to <4.6 ng/L | **Quartile 3**  4.6 to <6.2 ng/L | **Quartile 4**  ≥6.2 ng/L |
| *FI, n (%)* | 50 (16.3) | 44 (16.1) | 61 (21.3) | 80 (28.4) |
| *Model 2* | Ref. | 1.17 (0.87-1.56) | 1.33 (0.96-1.84) | **1.67 (1.12-2.50)** |
| *Fried phenotype, n (%)* | 12 (3.9) | 16 (5.9) | 20 (7.0) | 26 (9.2) |
| *Model 2* | Ref. | **2.06 (1.07-3.96)** | **2.34 (1.12-4.89)** | 2.16 (0.98-4.75) |
| ^1^Estimated odds and 95%CI from logistic regression analysis comparing the median hs-cTnI level from each quartile (Q) compared to Q1. Median Q1, Q2, Q3 and Q4 for hs-cTnI was 3.2, 4.2, 5.3 and 8.5 ng/L, respectively. Model 2: adjusted for age, LCN2, smoking history, socioeconomic position and alcohol intake. Bolded indicates p<0.05 compared to Q1. | | | | |

| **Table S4.** Odds ratio (95%CI) for the presence of frailty, derived from either the Frailty Index (FI) or modified Fried phenotype, by quartiles of high-sensitivity cardiac troponin-I (hs-cTnI) in the multivariable-adjusted model with the addition of total protein intake (g/d). | | | | |
| --- | --- | --- | --- | --- |
|  | **Quartiles of hs-cTnI^1^** | | | |
|  | **Quartile 1**  <3.8 ng/L | **Quartile 2**  3.8 to <4.7 ng/L | **Quartile 3**  4.7 to <6.3 ng/L | **Quartile 4**  ≥6.3 ng/L |
| *FI, n (%)* | 50 (16.3) | 44 (16.1) | 61 (21.2) | 80 (28.4) |
| *Model 2* | Ref. | 1.18 (0.88-1.58) | **1.38 (1.00-1.90)** | **1.80 (1.21-2.68)** |
| *Fried phenotype, n (%)* | 12 (3.9) | 16 (5.8) | 20 (6.9) | 26 (9.2) |
| *Model 2* | Ref. | **2.11 (1.09-4.07)** | **2.48 (1.18-5.19)** | **2.44 (1.12-5.34)** |
| ^1^Estimated odds and 95%CI from logistic regression analysis comparing the median hs-cTnI level from each quartile (Q) compared to Q1. Median Q1, Q2, Q3 and Q4 for hs-cTnI was 3.2, 4.2, 5.3 and 8.5 ng/L, respectively. Model 2: adjusted for age, total protein intake, smoking history, socioeconomic position and alcohol intake. Bolded indicates p<0.05 compared to Q1. | | | | |


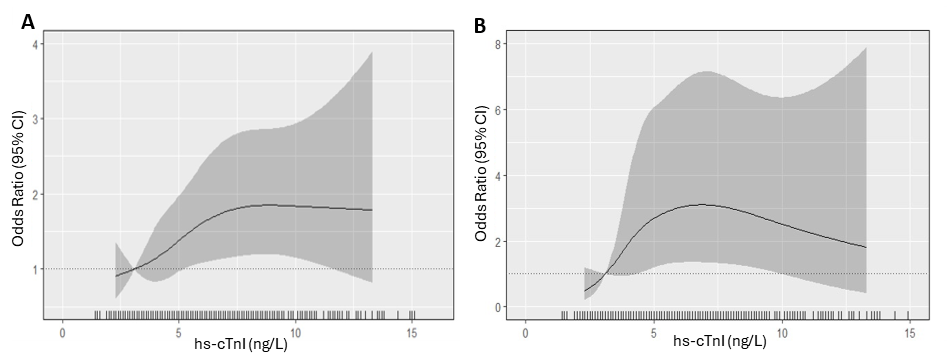


**Figure S1** Odds ratios from multivariable-adjusted logistic regression models with restricted cubic spline curves describing the association between high-sensitivity cardiac troponin-I (hs-cTnI) and the presence of frailty, based upon either the Frailty Index (A) or modified Fried phenotype (B), in 1101 individuals with hs-cTnI <15.6 ng/L. Odds ratios are based on models adjusted for age, smoking history, socioeconomic position and alcohol intake (Model 2). The odds ratio compares hs-cTnI levels (horizontal axis) to the median hs-cTnI level of the lowest quartile (3.1 ng/L). Shading represents 95% confidence regions. The rug plot along the bottom of each graph depicts an observation.
